# Supplementary material for: RNase A Domain-Swapped Dimers Produced Through Different Methods: Structure–Catalytic Properties and Antitumor Activity
Source: Life (Basel). 2021 Feb 21;11(2):168. doi: 10.3390/life11020168 (PMC7926746; doi:10.3390/life11020168)
Supplement: Supplementary file 1 [file life-11-00168-s001.pdf]

life

MDPI

Supplementary Material

RNase A Domain-Swapped Dimers Produced Through Different Methods: Structure–Catalytic Properties and Antitumor Activity

Riccardo Montioli [1], Rachele Campagnari [1], Sabrina Fasoli [1], Andrea Fagagnini [1], Andra Caloiu [2], Marcello Smania [1], Marta Menegazzi [1,*] and Giovanni Gotte [1,*]

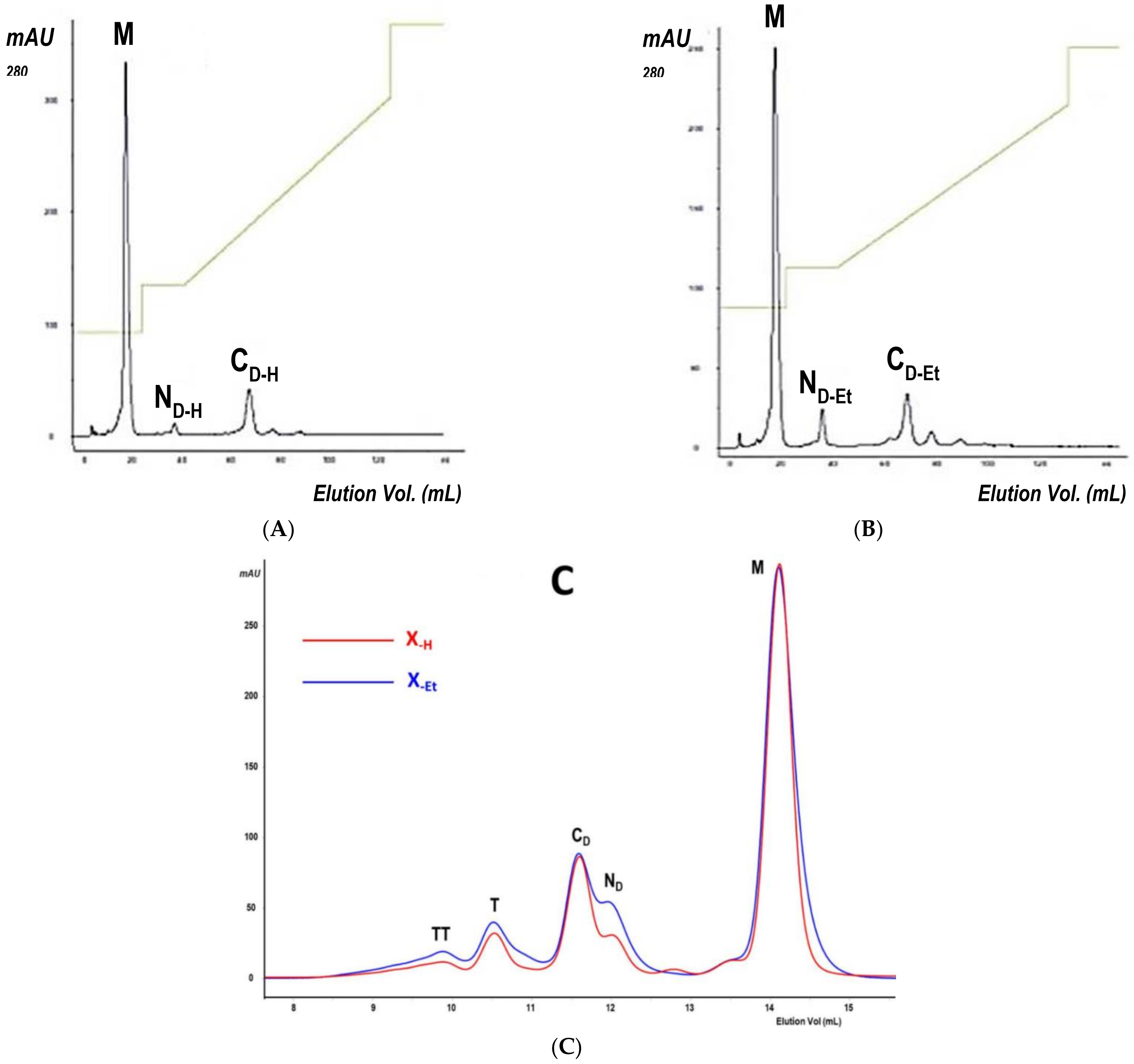

Figure S1. RNase A oligomers purification. **(A & B)** Cation Exchange purification performed in a Source 15S HR 10/10 column [1] of RNase A oligomers forming from **(A)** 40 % HAc-lyophilization (3), or **(B)** 40% EtOH 2 h incubation at 60 °C (4). The green line reports the[NaPi] increase from 70 to 90 mM and the linear gradient from 90 to 180 mM; **(C)** Overlayed SEC patterns of the same RNase A oligomerization deriving from HAc lyophilization (red curve, X_{-H} species), or 40%

EtOH thermal incubation (blue, X_{-Et} species), in a Superdex 75 Increase HR 10/300 column equilibrated with 0.2 M NaPi, pH 6.7. RNase A monomer, N_D and C_D (dimers), trimers (T) and tetramers (TT) are indicated in all panels.

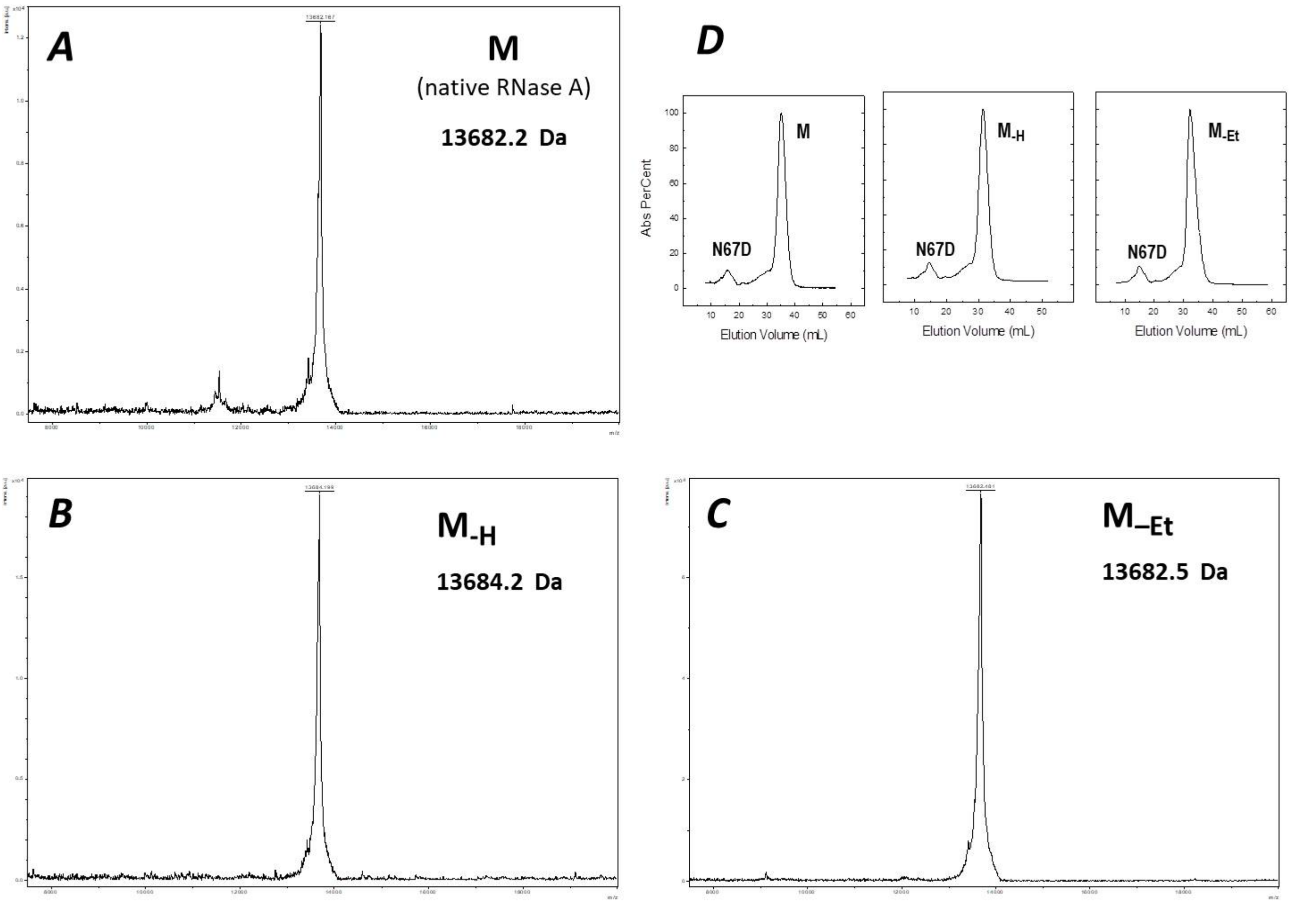

Figure S2. – Mass Spectrometry (MS) and deamidation analyses of RNase A monomers. **(A-C)** MS spectra of native RNase A M and of M_{-H} and M_{-Et}. Each relative M.W. value is reported; **(D)** Cation exchange chromatographic analysis of the deamidation of M, M_{-H} and M_{-Et} following the conditions indicated in (*26*). The positions of native M and of the mono-deamidated N67D-derivative are indicated.
